# Reducing violence and aggression: a quality improvement project for safety on an acute mental health ward

**DOI:** 10.1136/bmjoq-2023-002448

**Published:** 2023-12-28

**Authors:** Katrina Kernaghan, Kay Hurst

**Affiliations:** 1 Department of Health Professions, Manchester Metropolitan University, Manchester, UK; 2 Greater Manchester Mental Health NHS Foundation Trust, Manchester, UK

**Keywords:** Quality improvement, Mental health, Nurses

## Abstract

Violence and aggression (V&A) are identified as an issue on mental health wards that negatively affect staff, patients, care delivery and safety. This project took place on a male acute mental health ward where V&A are known to be an issue with an average of 21.3 incidences per month in the 6 months preceding the project.

The aim was to use QI (Quality Improvement) methodology to reduce incidences of V&A by 20% over a 4-month period. A root cause analysis was completed with staff, previous QI projects and literature on interventions for V&A were reviewed. Two changes were introduced via PDSA (plan, do, study, act) cycles; first was a safewards bundle including a training package, weekly emails and noticeboard displays, the second was safety crosses displayed on the ward.

There was a reduction in incidences of V&A across the project, mean weekly incidences reduced from 2.5 at baseline audit to 2.0 at the end of the project. This equates to a 20% reduction in V&A. The project did result in an increase of safewards interventions recorded and staff ratings of ward safety improved. There was a statistically significant correlation found between incidences of V&A and rates of restrictive practices.

Further analysis of the 20% reduction did not find a special cause variation, so results may be due to a common cause variation rather than the QI interventions. Safety crosses were not found to have an impact on rates of V&A, it is likely these need to be more embedded into communication about V&A on the ward. Potential confounding patient variables such as illicit drug use and history of V&A as well as staffing should be recorded and monitored in future projects. Recommendations to enhance further change should include regular meetings with both staff and patients to support open communication about the topic.

**WHAT IS ALREADY KNOWN ON THIS TOPIC**

- Violence and aggression (V&A) are a problem on mental health wards resulting in negative effects for staff and patients. There are interventions that are known to reduce V&A, shown by previous randomised control trials and QI projects.

**WHAT THIS STUDY ADDS**

- This QI project found implementing changes on a ward level with front line staff can result in more interventions being delivered and can make staff feel safer. To further embed the changes and increased open communication with staff and patients is needed with some monitoring of other variables.

**HOW THIS STUDY MIGHT AFFECT RESEARCH, PRACTICE OR POLICY**

- Using QI methodology at a ward level can positivley impact practice. This does not necessarily require systemic wide changes but empowering staff to use already known interventions to reduce V&A can improve the ward environment.

## Problem

It has long been known that violence and aggression (V&A) are an issue within mental health inpatient settings.^1–3^ Rates of V&A have been reported from 47% up to almost 100% for registered mental health nurses.^4–8^

The project was undertaken at one site, on a 20 bedded male mental health acute ward within Greater Manchester Mental Health NHS Foundation Trust. The ward is made up of a ward manager, charge nurses, staff nurses, a support time recovery worker, an assistant practitioner, support workers and a medical team. Patients are admitted voluntarily or detained under the mental health act (MHA) 9 for assessment and treatment of mental health problems. Problems with V&A can be exacerbated when caring for someone who is detained against their will. Admission criteria is often based on risk (to self, others or of vulnerability) assessed as too high to manage in a community setting.

Epidemiological studies of violence and serious mental illness have shown low risk for violence associated with serious mental illness unless there is a comorbidity with illicit substance misuse.^10 11^ For Manchester hospital admissions where drug-related mental and behavioural disorders were a factor the rate is almost double the national average at 183%.^12^

V&A were a problem on the project ward, in the preceding 6 months to the QI project the average rate of V&A incidents was 21.3 per month. The Trust has a Safe Working Policy detailing commitment to prevention and management of V&A from service users towards staff. The aim of this project was to reduce the rates of V&A on the ward by 20% over a 4-month period.

## Background

As V&A are known to be an issue, details of impacts and what may help need to be considered. Effects of V&A for staff and patients can be detrimental for safety, quality of care and delivery of care. These include poorer patient outcomes, negative psychological outcomes, physical injuries and reduced job satisfaction.^4 6 13–16^ This can result in increased reliance on agency staff, therefore the level of trained nurses on acute wards has fallen due to higher staff turnover rate. This can have a knock-on effect on patient care.^17^ There are also financial costs associated with assaults, verbal abuse and damage to property with an estimated annual cost of £20.5 million per year in psychiatric wards in England,^18^ this can result in more acute service use including internal transfers and increased occupied bed days.^19^

There are a range of methods or techniques used to manage V&A on psychiatric wards, including talking to the patient, increasing observation levels, oral PRN (when required) medication, rapid tranquilisation (RT) medication given intramuscularly, physical restraint and seclusion (where patients are secluded in a locked room for containment of severe behavioural disturbance likely to cause harm to others, MHA code 2015). On occasion restrictive practices (defined here as RT medication, physical restraint and seclusion) are necessary for safety of both staff and patients. However, they carry risks including physical harm to staff and patients, can result in staff experiencing cognitive dissonance and anxiety, feelings of anger and fear, with a negative impact on dignity.^6 20–22^

Interventions for this QI project were developed after a literature review of V&A on mental health wards including a randomised control trial (RCT) on the ‘Safewards Model’^23^ and reviewing other QI projects on V&A on mental health wards.^24 25^ A root cause analysis (RCA) with various stakeholders was also completed.

The safewards model RCT aimed to reduce frequency of conflict and containment (threats to safety and acts to minimise harm) across 31 inpatient wards. Alternative ways nursing staff could respond to potential V&A without using containment or restrictive practice were tested. Ten interventions were identified as the best way to create a positive ward environment that maximised collaboration and communication with tools for prevention and de-escalation. Results found safewards interventions reduced conflict by 15% and containment by 26%. This RCT population of an inner city acute mental health ward is generalisable to the QI project ward.

There are case studies of good practice in UK and Australian hospitals that have adopted safewards, demonstrating applicability and benefits in real life settings. Reviews concluded safewards provide a tested, holistic framework to improve the overall ward environment and communication between staff and patients.^26^ Systematic reviews of safewards found reductions in incidences of V&A and improvement in safety experience of staff when combined with training and recommended safewards be widely used.^27 28^

Two QI projects aiming to reduce violence on mental health inpatient units were reviewed, they used the same QI methodology as this project with PDSA cycles to test ideas for change. One QI project^25^ used an interventions test bundle (including a structured risk assessment for violence, safety crosses displayed on wards, safety huddles and discussions in ward community meetings) using PDSA cycles over a 15-month period. A 40% reduction in physical violence across all six wards was achieved. This demonstrates how QI can result in positive changes in V&A that were hoped to be replicated in this project.

Another QI project^24^ tested a change bundle including safety crosses, safety huddles and weekly community safety discussions. PDSA cycles were used over an 18-month period and an 8% reduction in physical violence was achieved and sustained. The QI approach was noted to be effective at reducing V&A, brought improvements and a cultural shift towards openness around violence. Examining these previous works enabled this project to plan change interventions based on what has worked in the past.

## Measurement

The primary outcome measure was incidents of V&A. This included physical abuse or violence by patients towards staff or other persons and physical aggression to property. To support validity verbal aggression, financial, sexual or racist abuse were not included. Incidences of V&A are reported via electronic incident reports, and data collected from these each week.

The process measures were the percentage of staff that completed the safewards training bundle and recording of safewards interventions on electronic notes. This allowed the project lead to monitor if the change measures were actually being put in place, it was expected that safewards interventions recorded would increase showing they were being more frequently implemented.

The balancing measures were staff ratings of ward safety at the start, middle and end of the project and rates of restrictive practices. Monitoring these allow demonstration of knock on effects of any improvement. Staff’s rating safety on the ward is important for morale and effective care. If staff feel unsafe their approach to work will likely be negatively affected as well as impacting their psychological well-being.^13 14^ There were some foreseen potential issues with reliability of the ward safety ratings, as responses may depend on how acute the ward was at the time (eg, potential staff may rate the ward more unsafe if there had been an incident that day compared with a day with no incidents). So ratings were done as close together as feasible, but not possible to do them all on 1 day as only certain staff worked on each shift.

## Design

### Patient and public involvement

Patients were not involved in the design of the safewards bundle as the safewards model was pre-existing and had previously been developed with patient involvement. Patients as stakeholders were engaged in collaborative discussions around safety crosses that were displayed on the ward detailing incidences of V&A.

Initially, an RCA was completed to discover physical, latent and human factors affecting V&A on the ward. RCA allows the source of the issue to be identified so resources for QI can be appropriately directed towards the problem rather than the symptoms.^29^ This included stakeholder meetings, development of a fishbone and driver diagrams with a range of staff including ward staff, the senior leadership team, the unit psychologist and medical staff. The need for the project was evident from these.

QI methodology using PDSA cycles to test change ideas was used, although the time scale for this project was significantly less than other QI studies reviewed,^24 25^. This had impact on consideration of interventions, as well as staff burden and what would be most effective in a short space of time.

### Interventions

Two interventions identified to be used in this QI project were: safewards training bundle and safety crosses displayed on the ward.

The staff team are known to be influential with the greatest control over the physical and psychosocial quality of the ward environment, interventions that enhance staff modifiers can reduce conflict and containment. 23 This led to the current QI project lead developing a training package around four safewards interventions; calm down methods, talk down, soft words and bad news mitigation. Considering the timelines of the two QI projects reviewed^24 25^ were much longer than the current project, only certain aspects of the change interventions were included to test initially. The specific interventions were selected as they are applicable tools to use when face to face with patients, rather than something systemic requiring change of the system which they may feel they have less control over (such as arranging daily meetings for the whole ward, staffing numbers). Empowering front-line staff was considered very important to make the QI project successful. Implementation of the safewards model is supported by the Trust’s Positive and Safe Strategy and NICE (National Institute for Health and Care Excellence) guidelines.^30^ It was important to provide staff with tools to help and empower them during face-to-face interactions with patients and that change interventions were applicable and practical.

Staff and patient awareness and communication about V&A on the ward was important to keep momentum and involve patients, so the second intervention tested via PDSA cycle two was safety crosses displayed. Another factor in selecting these was they did not result in more paperwork for staff (such as a violence assessment checklist or huddle meetings) to avoid any burden on their time.

## Strategy

The aim of the project was to reduce incidences of V&A by 20% over a 4-month period. An initial audit period collected baseline data, then the first PDSA cycle for 4 weeks, an audit period for 4 weeks, the second PDSA cycle for 4 weeks and then a final 4-week audit period.

### PDSA cycle 1 - Intervention 1: Safewards Bundle

This took place over 4 weeks had three aspects: (1) 1-hour training session on four safewards interventions (soft words, talk down methods, bad news mitigation, calm down methods) delivered to nursing assistants and nurses. This included a PowerPoint presentation, handouts and safeward video clips. (2) Theme of the week notice boards. Displayed in the MDT (multidisciplinary team) room where handovers and ward rounds are completed. Each week a different intervention was the focus of the display. (3) Theme of the week email. These were sent to all staff weekly, containing an explanation of the intervention, with a link to safewards video or attached documents with information on the intervention.

There were 10 training sessions, designed and delivered by the project lead, completed twice per week at handover time when the manager would be able to provide cover on the ward. A separate session was arranged for night staff and a mop up session towards the end of the cycle for staff who had not been able to attend other sessions.

Data was gathered 4 weeks after PDSA cycle 1 and an increase in safewards interventions was observed and also a reduction in incidences of V&A.

### PDSA cycle 2 - Intervention 2: Safety Crosses

Sharing data with staff and patients can facilitate discussion and change. The second intervention was the safety crosses displayed on the ward showing incidences of V&A; green days when there were no incidences of V&A and red days when there were incidences. This would be a timely and simple way to record incidents and communicate this. There was also a box for how many days since the last incident. These were placed on the wall as you entered the ward, opposite the medication clinic so patients and staff regularly walked past. This was updated every 2 days over the 4 week period.

**Figure 1:**
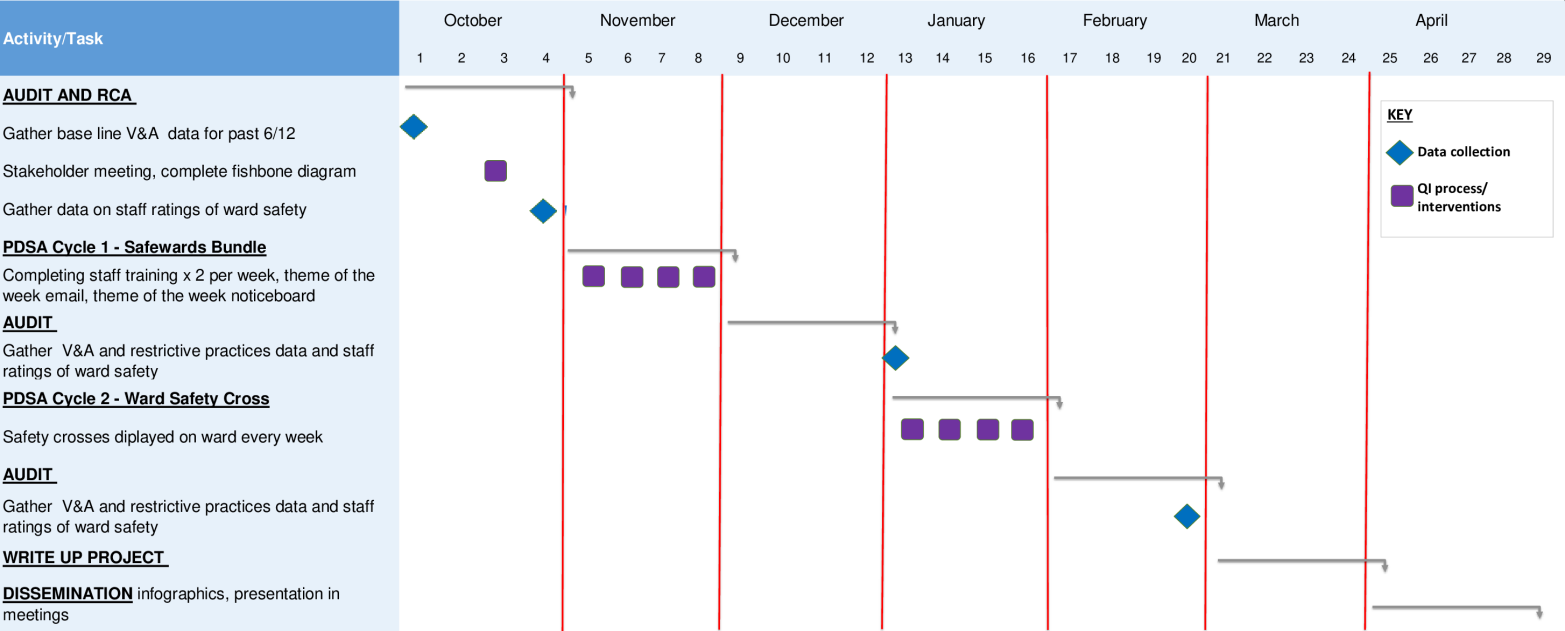
Violence and aggression reduction project: Gantt chart.

After PDSA cycle 2, another 4-week audit period continued to collect data (see figure 1).

## Results

**Figure 2:**
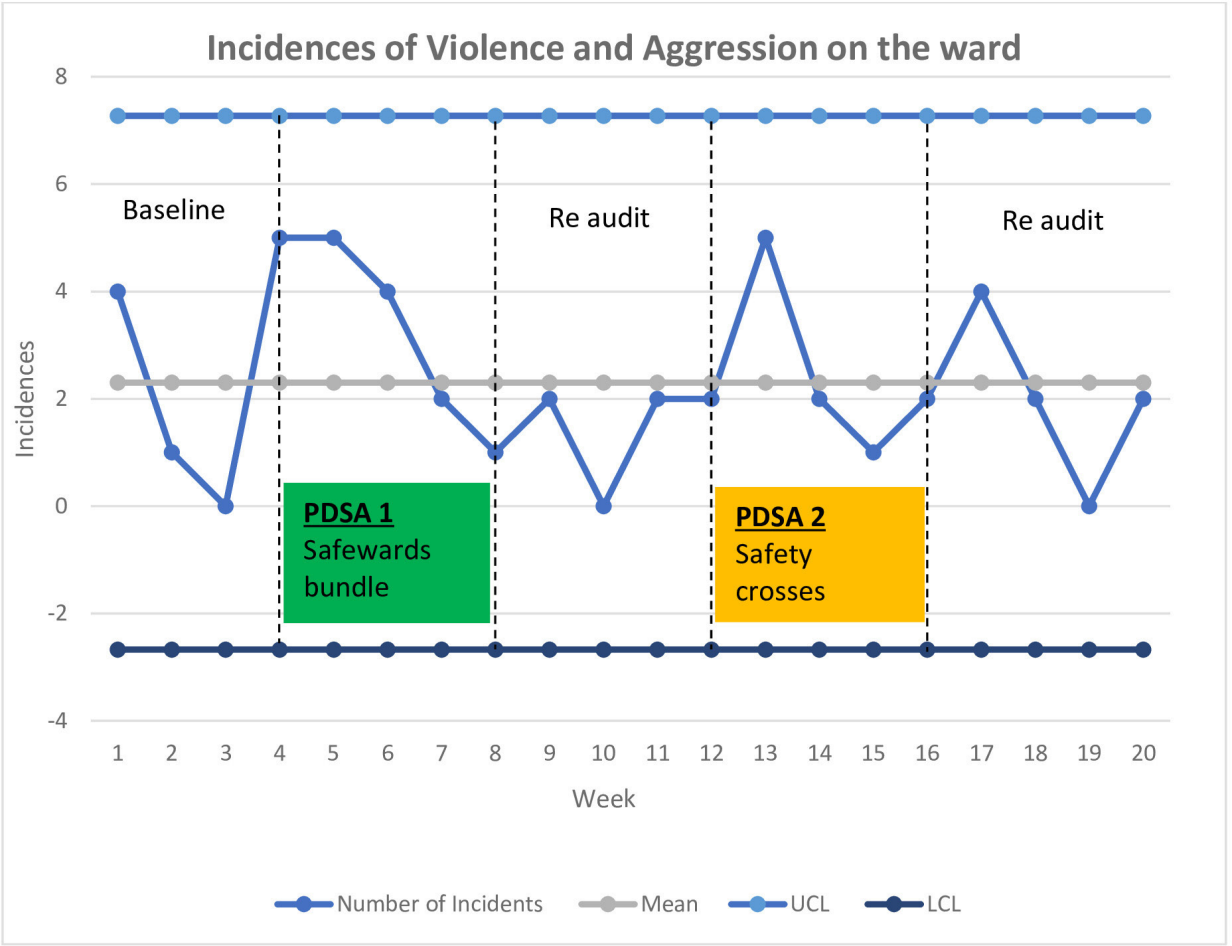
Incidences of violence and aggression on the ward across the project. UCL, upper control limit; LCL,Lower control limit.

Across the 20-week duration of the whole project a total of 46 incidents of V&A occurred, an average of 2.3 per week. There was a reduction in the incidences of V&A from 2.5 per week at baseline to 2.0 per week at final reaudit. This equates to a 20% reduction in V&A which met the aim. However, further analysis of the data did not find a special cause variation, so results may have been due to a common cause variation and not the QI interventions (see figure 2).

**Figure 3:**
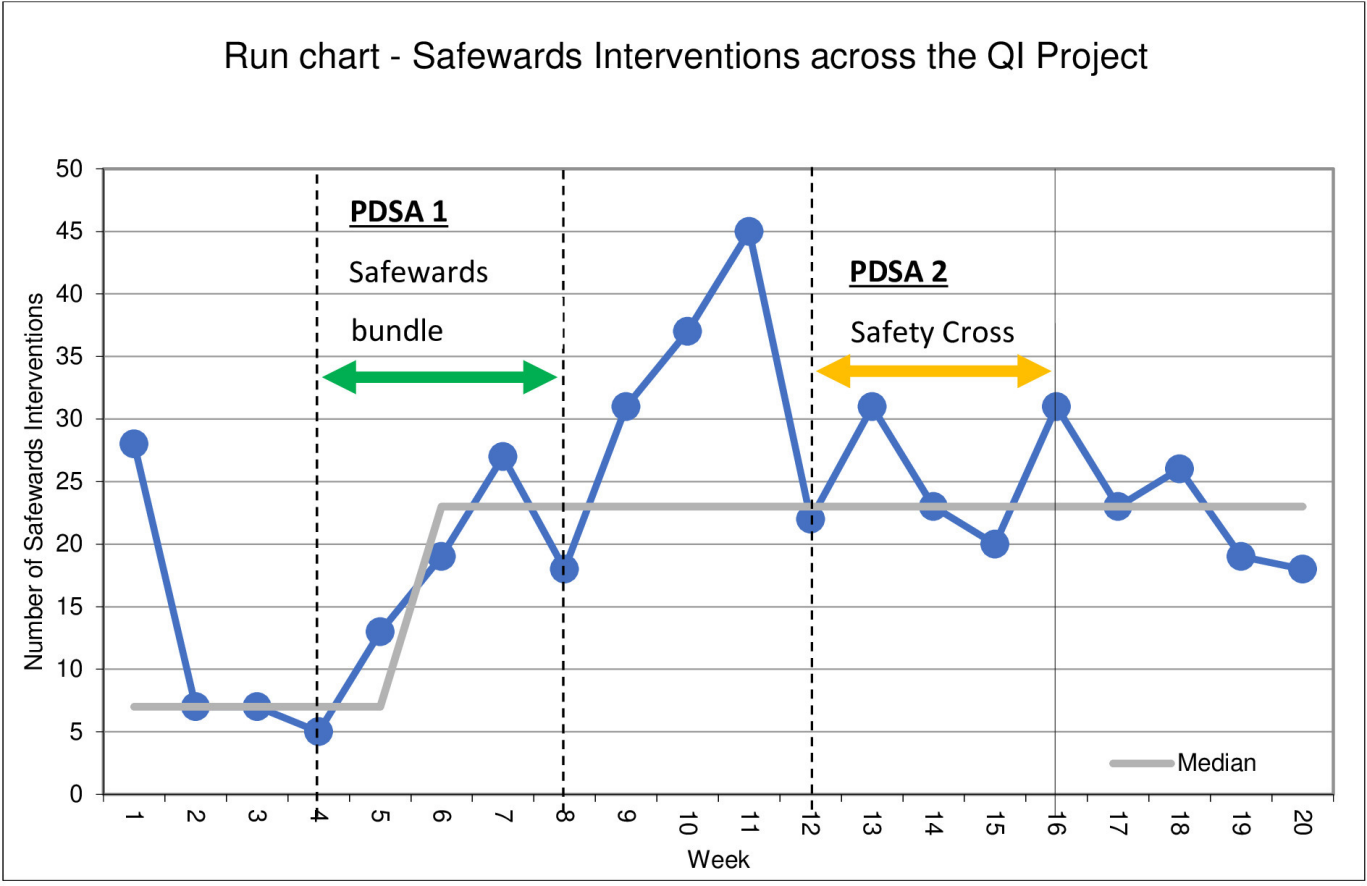
Run chart: recorded safewards interventions across the project.

There was an increase in the recordings of safeward interventions across the project with a baseline mean of 2.9 per week and 5.4 at the final reaudit. There was peak of 8.4 in the audit immediately after PDSA cycle 1 safewards bundle. Recording of safewards interventions was plotted on a run chart, there was one special cause variation identified, as there was a shift noted (see figure 3). This variation can be attributed to the safewards bundle PDSA cycle as it occurred during this cycle and during the reaudit period immediately after.

Staff rating of feeling ‘safe’ improved over the course of the project from 7% initially to 27% at the end. The percentage of staff feeling unsafe reduced during the project from 60% to 36% at the end.

There was little change in the mean number of restrictive practices across the project. Spearman’s correlation was completed to assess the relationship between incidences of V&A and restrictive practices, and a positive correlation found (r(18)=0.59, p=0.01). So as rates of V&A increase so did rates of restrictive practices. This was statistically significant correlation at the 0.01 level, so chances this is due to random error alone is less than 1%. No correlation was found between incidences of V&A and safewards interventions (r=0.05).

**Figure 4:**
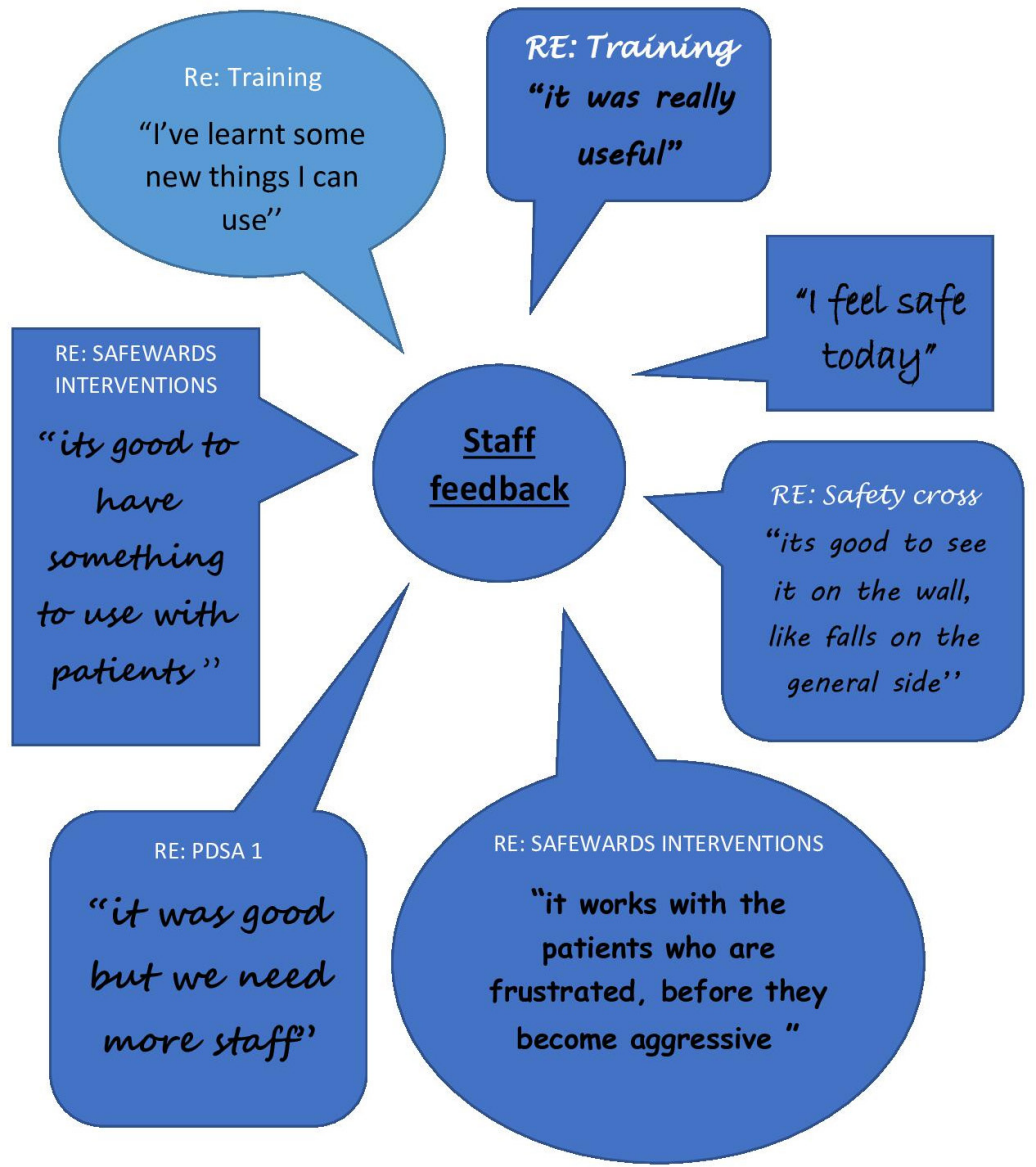
Staff feedback diagram.

Staff feedback on the QI project was generally positive (see figure 4). There was a good response to the safewards training, multiple staff reported finding it useful and one asked for more information. However, there was limited feedback around the safety cross use.

## Lessons and limitations

The reduction in V&A incidences was in keeping with previous findings.^23 26–28^ However, studies with reductions in V&A directly attributable to safewards interventions were longer in duration or more intense, for example, implementation of all ten safewards interventions over a 3- month period resulting in a smaller reduction in V&A.^23^ Despite being relatively short in duration compared with other QI projects improvements were seen in documented safewards interventions that coincided with PDSA cycle 1 indicating the safewards bundle did have an impact. This demonstrates engaging staff around interventions known to be effective can result in an increase in these, which has benefits for staff and patients. Moving forward projects would have greater impact by including all interventions. It is unclear if the outcome of this project would be applicable to other acute wards however, safewards interventions have been used successfully on female wards and in other countries.^31 32^

The project lead worked on the project ward and delivered the training which may have produced a bias, for example, the presence of project lead may have acted as a reminder of the interventions rather than this being due to safewards bundle alone. This Hawthorne effect could have impact on longer term sustainability once the project is over.

A strength was an improvement in how safe staff felt, feeling safe increased by 20% and there was a reduction in 25% of those who felt unsafe. This coincided with incidences having decreased by the end of the project and can potentially result in improved job satisfaction and reduction in negative psychological effects.^4 6 14^ Qualitative feedback showed staff found the safewards bundle helpful, feeling engaged and ultimately safer. A deeper understanding could be gained in future projects by using smartphone technology to gather information from staff as used in other recent studies.^33^

There was little impact of safety crosses, despite them being influential in other QI studies^24 25^ where they were used alongside other interventions that supported communication (eg, safety huddles, ward meetings) and for longer. On reflection the safety crosses alone as a change intervention were not enough to communicate the data to facilitate change. Sometimes violence is the elephant in the room^25^ and effective prevention needs a community culture.^34^ Therefore, future projects would use existing meetings with patients and staff to actively facilitate discussion and promote change.

Staffing is an issue highlighted during discussions around V&A and safety, there were attempts to mitigate this when planning the training delivery for example, doing this during handover periods when more staff were around. However, there were issues around ward acuity and V&A occurring when training was planned which resulted in 5% of staff being unable to complete the training. Also when the ward was understaffed, staff from other wards or agencies worked on the ward, but did not receive the safewards bundle or have any awareness of the project which may have affected results. Staffing issues are known within the NHS, the current project was unable to avoid or control for this issue. However, in previous studies^23^ staffing was taken into account (could only participate if had permanent ward manager, less than 30% vacancies) and could be a variable that impacts so should be monitored in future projects.

A limitation potentially affecting internal validity of the findings could be the data collection method (using electronic incident forms), for example, if three incidences occurred during a shift, only one report may be completed summarising all of these, resulting in under reporting. Also, if an incidence of V&A is considered by nurses to be minor with no one hurt, it is less likely to be reported in an electronic form. This is influenced by acuity of the ward, with time and limited resources being factors. Two options moving forward to address this are—staff to complete a tick list on the wall in the office when incidences occur, or the project lead accessing full electronic reports to further extrapolate data.

Due to the complex and multifaceted issue of V&A, some results found may be difficult to interpret, for example, numbers of restrictive practices did not show any change during the project. However, this may be because staff used these practices pre-emptively to successfully prevent V&A or as the rates were so low anyway (average of 0.56 across the whole project) so there was little range for further reduction. Correlation analysis found the rates of V&A and restrictive practices were positively correlated, and this was statistically significant. Often restrictive practices cannot be avoided when V&A occurs, for example, if one patient physically attacks another person a recourse of action is to use physical restraint.

There are also unmeasured confounding variables that could have affected the outcomes. Patient factors affecting V&A on mental health wards include admission under the MHA,^35 36^ younger age^6 37^ and illicit drug use.^6 38 39^ A variable strongly correlated with V&A is history of aggression^40 41^ and a recent study found 4% of service users accounted for 50% of V&A.^19^ Epidemiological studies of violence and serious mental illness have shown low risk for violence associated with serious mental illness unless there is a comorbidity with illicit substance misuse.^10 11^ For Manchester hospital admissions where drug-related mental and behavioural disorders were a factor, the rate is almost double the national average at 183%.^12^ Therefore, patient characteristics on the ward should be monitored during future projects to deepen understanding of the issue.

## Conclusion

V&A are an issue on inpatient wards with negative impacts on staff and patient care. The current project was partially successful, the aim was met with a decrease in V&A incidents by 20%, although not able to be attributed specifically to the interventions in the PDSA cycles. Improvements included an increase in safewards interventions recorded, this was found to be due to the safewards bundle PDSA cycle. Staff rating of ward safety improved over the project. This project shows small improvements can be made on a ward level that have positive impacts. A longer more sustained period of input around V&A is needed, improvements were beginning but as demonstrated by previous QI projects in this area it may require more than 12 months to embed.

At a ward level sustaining the project may be challenged by a tendency to relapse into old ways once the project is finished and new staff will have not had the training. To sustain changes there could be a brief training package as part of local ward induction for new starters and a ‘safewards’ notice board to be maintained and regularly updated. Another aspect for ongoing promotion could be the identification of safewards ‘champions’ recommended by safewards.net to drive the implementation of each intervention which has shown to work well.^42^

Next steps could be using staff and service user ward meetings to discuss V&A and promote change while monitoring other potential confounding variables (e.g, patient factors, staffing levels). If a reduction in V&A rates is demonstrated with a special cause variation in future PDSA cycles, there would be an opportunity for roll out to the wider unit.

## Data Availability

Data are available on reasonable request. Data available.
